# Concentration and Risk Evaluation of Polycyclic Aromatic Hydrocarbons in Urban Soil in the Typical Semi-Arid City of Xi’an in Northwest China

**DOI:** 10.3390/ijerph15040607

**Published:** 2018-03-27

**Authors:** Lijun Wang, Shengwei Zhang, Li Wang, Wenjuan Zhang, Xingmin Shi, Xinwei Lu, Xiaoping Li, Xiaoyun Li

**Affiliations:** 1 Department of Environmental Science and Engineering, School of Geography and Tourism, Shaanxi Normal University, Xi’an 710119, China; SWZ0112@126.com (S.Z.); budingmei@snnu.edu.cn (L.W.); zwj062060@163.com (W.Z.); realsimon@163.com (X.S.); luxinwei@snnu.edu.cn (X.Lu.); lixiaoping@snnu.edu.cn (Xp.L.); lixiaoyun518@163.com (Xy.L.); 2 International Joint Research Center of Shaanxi Province for Pollutant Exposure and Eco-Environmental Health, Xi’an 710062, China

**Keywords:** polycyclic aromatic hydrocarbon, ecological risk, health risk, urban soil

## Abstract

Polycyclic aromatic hydrocarbons (PAHs) are ubiquitous environmental pollutants, presenting potential threats to the ecological environment and human health. Sixty-two urban soil samples were collected in the typical semi-arid city of Xi’an in Northwest China. They were analyzed for concentration, pollution, and ecological and health risk of sixteen U.S. Environmental Protection Agency priority PAHs. The total concentrations of the sixteen PAHs (Σ16PAHs) in the urban soil ranged from 390.6 to 10,652.8 µg/kg with an average of 2052.6 µg/kg. The concentrations of some individual PAHs in the urban soil exceeded Dutch Target Values of Soil Quality and the Σ16PAHs represented heavy pollution. Pyrene and dibenz[a,h]anthracene had high ecological risk to aquatic/soil organisms, while other individual PAHs showed low ecological risk. The total ecological risk of PAHs to aquatic/soil organisms is classified as moderate. Toxic equivalency quantities (TEQs) of the sixteen PAHs varied between 21.16 and 1625.78 µg/kg, with an average of 423.86 µg/kg, indicating a relatively high toxicity potential. Ingestion and dermal adsorption of soil dust were major pathways of human exposure to PAHs from urban soil. Incremental lifetime cancer risks (ILCRs) of human exposure to PAHs were 2.86 × 10^−5^ for children and 2.53 × 10^−5^ for adults, suggesting that the cancer risk of human exposure to PAHs from urban soil is acceptable.

## 1. Introduction

Cities are the densest area of anthropogenic activities [1,2]. Owing to the rampant development of industrialization, urbanization, and agricultural modernization, urban soil, as an important part of urban ecosystems, has been suffering serious contamination with various pollutants, such as heavy metals, polychlorinated biphenyls, phthalate esters/phthalic acid esters, and polycyclic aromatic hydrocarbons (PAHs). These harmful pollutants accumulated in urban soil can be carried into surface/ground water through precipitation and urban runoff, emitted into atmosphere by volatilization, and transported into crops from polluted soil and air via root and leaf adsorption, which may indirectly result in further water, atmosphere, and food pollution [3,4,5]. They can also be transferred to the human body via ingestion, inhalation, and dermal adsorption of soil dust, which has a direct influence on human health, especially for children and elderly people who are physiologically more vulnerable to environmental contamination [6]. Therefore, urban soil has become a main reservoir of various pollutants and a transmitter of pollutants to water bodies, atmosphere, crops, and human beings; its quality is also a valid indicator of pollution and environmental risks [2,3,4,5,7,8,9,10,11,12,13,14,15,16].

PAHs consisting of two or more fused benzene rings are widespread in water, air, soil, dust, and sediment. Their natural sources in the environment include volcanic eruptions, forest fires, diagenesis and biosynthesis [2,17]. The predominant anthropogenic source is incomplete combustion of organic substances, such as coal, petroleum, natural gas, wood, grass, straw, and tobacco [2,17,18,19,20,21,22,23,24]. They are characterized by their high toxicity as well as the potential effects of carcinogenicity, teratogenicity, and mutagenicity, which are associated with human health, such as cataracts, kidney/liver damage, and jaundice [18,24]. Therefore, the U.S. Environmental Protection Agency (USA EPA) [25] has classified sixteen PAHs as priority pollutants. Meanwhile, USA EPA [25] and the International Agency for Research on Cancer [26] have also considered seven of sixteen priority PAHs as probable/possible human carcinogens. In addition, they are considered as candidates of persistent organic pollutant (POP) that merit further investigation for possible early inclusion into the Stockholm Convention on POPs [27]. Thus, more attention has been paid to PAHs in recent years.

Xi’an is not only the capital of Shaanxi Province and the largest city in Northwest China, but also a typical semi-arid city. It suffers from serious environmental pollution problems because of rapid industrialization and urbanization. Chen et al. [28,29] have conducted some studies on heavy metal pollution in urban soil in Xi’an. However, limited data on PAHs pollution in urban soil in Xi’an are available. Therefore, this study aims to determine the concentrations of PAHs in urban soil of Xi’an, assess the pollution level and ecological risk of PAHs to aquatic/soil organisms, and evaluate the toxicity and health risk of human exposure to PAHs from urban soil.

## 2. Materials and Methods

### 2.1. Description of Studied Area

Xi’an lies in the middle part of the Guanzhong Plain surrounded by the Qinling Mountains in the south and by the Loess Plateau in the north. It spans an urban area of some 1066 km^2^ with an urban population of 5.8 million [30]. The climate is characterized by large seasonal variations associated with the East Asian monsoons. The northerly Asian winter monsoon prevails in winter, transporting Asian dust from Chinese deserts and nearby loess area. The southeast monsoon brings moisture to the region in summer [31]. The annual air temperature is approximately 13 °C with an annual precipitation of 558–750 mm. Xi’an is also an important center of economy, education, culture, manufacturing, and high-tech industries in Northwest China. The sum of motor vehicles in Xi’an ranges from 0.52 million in 2003 to 1.86 million in 2013 [30]. Domestic heating by coal combustion in Xi’an occurs from November of a year to the next March.

### 2.2. Sample Collection and Pre-Treatment

**Figure 1:**
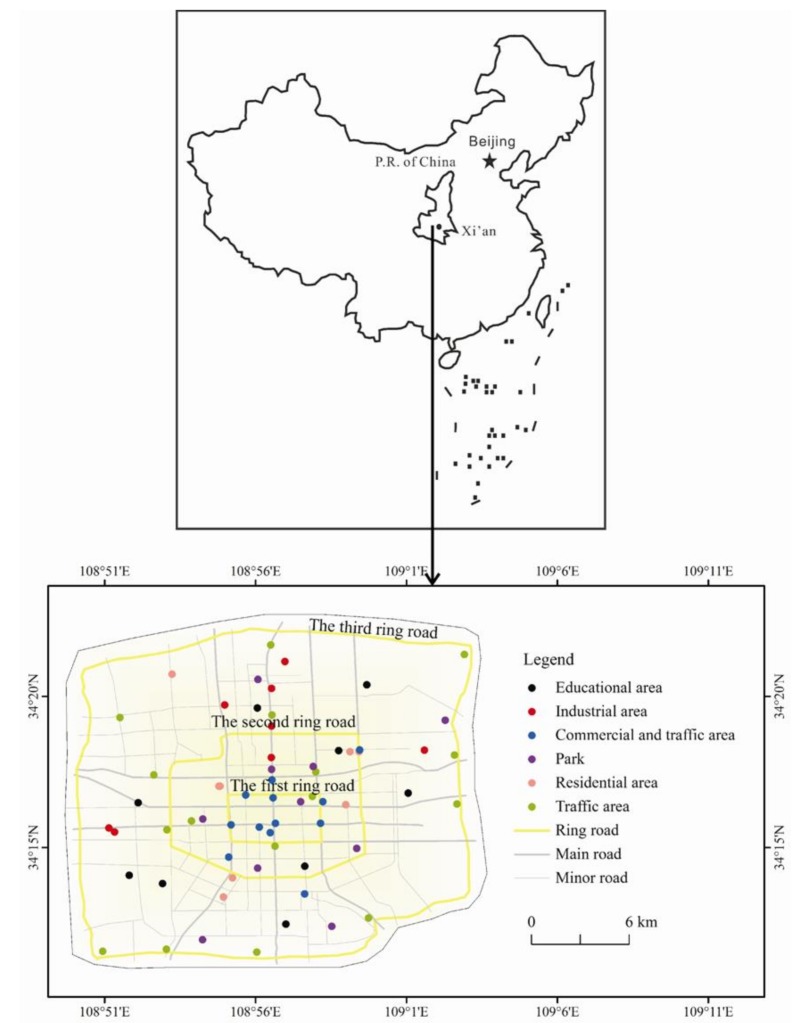
Location of study area and sampling sites of urban soil in Xi’an.

A total of sixty-two soil sampling sites were set up in Xi’an City in Northwest China (Figure 1), including six urban functional districts, i.e., industrial areas, traffic areas, mixed commercial and traffic areas, residential areas, educational areas, and parks. Five sub-topsoil samples (0–20 cm) were collected at each sampling site from the four corners and center in a 2 m × 2 m grid with a stainless steel shovel. They were mixed into a composite topsoil sample of ~1 kg by a quartile method on the spot. Each composite topsoil sample was stored in a brown glass bottle, and then taken back to laboratory. All collected topsoil samples were air-dried in a cool, dark, and ventilated place at room temperature. The air-dried topsoil samples were first crashed, then sieved through a 1 mm stainless steel sieve to remove small stones, plant debris and other refuses, and finally stored in brown glass bottles at 4 °C before analysis.

### 2.3. Analysis of PAHs

PAHs in urban soil were first extracted using a Soxhlet extraction apparatus with a solution of *n*-hexane and acetone (1:1, *v*:*v*), then purified by using a glass chromatography column of silica gel/neutral alumina (2:1, m:m), and finally separated at a fused silica capillary column (30 m × 0.25 mm × 0.25 μm, Alltech, Chicago, IN, USA) via an 7890A gas chromatograph (Agilent, Palo Alto, CA, USA) equipped with a flame ionization detector (GC-FID). The details of extraction, purification, and instrumental analysis as well as quality control and assurance were described in our previous studies [32,33]. The results of process blank experiments showed that PAHs were not detected in rinsates. The instrument detection limit (LDL) calculated as the ratio of three times signal to noise was 0.003 μg/mL for acenaphthylene (Acy), acenaphthene (Ace), fluorene (Flu), phenanthrene (Phe) and anthracene (Ant), 0.005 μg/mL for fluoranthene (Fla), 0.006 µg/mL for naphthalene (Nap) and pyrene (Pyr), 0.012 µg/mL for benzo[a]anthracene (BaA), chrysene (Chy), benzo[b]fluoranthene (BbF) and benzo[k]fluoranthene (BkF), 0.015 µg/mL for benzo[a]pyrene (BaP), 0.017 µg/mL for indeno[1,2,3-cd]pyrene (InP), 0.020 µg/mL for dibenz[a,h]anthracene (DBA), and 0.023 µg/mL for benzo[g,h,i]perylene (BghiP), respectively. The recovery of decafluorobiphenyl as a surrogate standard varied between 79% and 113% with an average of 102%. The recovery of matrix addition standard ranged 67% to 119%. Ten percent of urban soil samples were duplicated, and the relative standard deviation (RSD) was below 11%.

### 2.4. Ecological Risk Assessment

PAHs accumulated in urban soil may enter water bodies and plants, posing a potential ecological risk. Kalf et al. [34] proposed assessing ecological risk of some organic substances using a risk quotient (RQ). Cao et al. [35] improved the method by considering toxic equivalency factors. This improved method was used to assess the ecological risk of PAHs in the urban soil. The risk level posed by certain PAHs was characterized by the risk quotient (RQ), which was calculated with Equation (1):

(1) $$\mathrm{RQ}=\frac{C_{PAHs}}{C_{QV}}$$

where C_PAHs_ is the concentration of certain PAHs in soil and C_QV_ is the corresponding quality values of certain PAHs in soil. In the present study, the negligible concentrations (NCs) and the maximum permissible concentrations (MPCs) of PAHs in soil reported by Kalf et al. [34] were used as the quality values in soil. MPCs are the concentrations in the environment above which the risk of adverse effects is considered unacceptable to ecosystems, and NCs are the concentrations in the environment below which the occurrence of adverse effects is considered to be negligible [36]. Therefore, RQ_NCs_ and RQ_MPCs_ were defined as follows:

(2) $${\mathrm{RQ}}_{NCs}=\frac{C_{PAHs}}{C_{QV(NCs)}}$$

(3) $${\mathrm{RQ}}_{MPCs}=\frac{C_{PAHs}}{C_{QV(MPCs)}}$$

where C_QV(NCs)_ is the quality values of the NCs of PAHs in the medium and C_QV(MPCs)_ is the quality values of the MPCs of PAHs in the medium. The RQ_ΣPAHs_, RQ_ΣPAHs(NCs)_ and RQ_ΣPAHs(MPCs)_ is defined as follows:

(4) $${\mathrm{RQ}}_{\Sigma SPAHs}=\sum_{i=1}^{16}RQ_{i}RQ_{i}\geq 1$$

(5) $${\mathrm{RQ}}_{\Sigma PAHs(NCs)}=\sum_{i=1}^{16}RQ_{i(NCs)}RQ_{i(NCs)}\geq 1$$

(6) $${\mathrm{RQ}}_{\Sigma PAHs(MPCs)}=\sum_{i=1}^{16}RQ_{i(MPCs)}RQ_{i(MPCs)}\geq 1$$

The RQ_(NCs)_ and RQ_(MPCs)_ of individual PAHs which were not less than 1 were summated to calculate the RQ_ΣPAHs(NCs)_ and the RQ_ΣPAHs(MPCs)_ of total PAHs to fully consider the ecological risk of individual PAHs. The ecological risk classification is listed in Table 1. RQ_(NCs)_ < 1.0 indicated that the single PAHs might be of negligible concern, RQ_(MPCs)_ > 1.0 would indicate that the contamination of the single PAHs posed severe risk, and RQ_(NCs)_ > 1.0 and RQ_(MPCs)_ < 1.0 indicated that the contamination of the single PAHs was of moderate risk.

### 2.5. Health Risk Evaluation

USA EPA [37] has developed a standard model of cancer risk assessment, i.e., incremental lifetime cancer risk (ILCR). ILCR was widely used in many studies [24,38,39,40,41,42,43,44,45]. ILCR was used to quantitatively estimate the cancer risk of human exposure to PAHs in the environment. Humans can be exposed to PAHs in urban soil through ingestion, inhalation, and dermal adsorption of soil dust. Equations (7)–(9) were used to evaluate the ILCR of each exposure pathway:

(7) $$ILCRs_{Ingestion}=\frac{CS\times (CSF_{Ingestion}\times \sqrt[{3}]{\frac{BW}{70}})\times IR_{Ingestion}\times EF\times ED}{BW\times AT\times 10^{6}}$$

(8) $$ILCRs_{Inhalation}=\frac{CS\times (CSF_{Inhalation}\times \sqrt[{3}]{\frac{BW}{70}})\times IR_{Inhalation}\times EF\times ED}{BW\times AT\times PEF}$$

(9) $$ILCRs_{Dermal}=\frac{CS\times (CSF_{Dermal}\times \sqrt[{3}]{\frac{BW}{70}})\times SA\times AF\times ABS\times EF\times ED}{BW\times AT\times 10^{6}}$$

where CS is the total of toxic equivalency quantities (TEQs) of sixteen PAHs relative to BaP using the toxic equivalency factors (TEFs) listed in Table 7 below [46,47], the TEQ of certain PAH equals to its measured concentration times its corresponding TEF; CSF is the cancer slope factor, (mg/kg/day)^−1^, the CSFs of BaP were determined by the cancer-causing ability of BaP and were 7.3 (mg/kg/day)^−1^ for ingestion, 3.85 (mg/kg/day)^−1^ for inhalation, and 25 (mg/kg/day)^−1^ for dermal adsorption, respectively [41]; BW is body weight, kg; AT is the average life span, day; EF is the exposure frequency, day/year; ED is the exposure duration, year; IR_Ingestion_ is the soil intake rate, mg/day; IR_Inhalation_ is the inhalation rate, m^3^/day; SA is the dermal surface exposure area, cm^2^; AF is the dermal adherence factor, mg/cm^2^; ABS is the dermal adsorption fraction, unitless; PEF is the particle emission factor, m^3^/kg. The values of assessment parameters in this study were from U.S. EPA and related literatures (Table 2).

## 3. Results and Discussion

### 3.1. Concentration of PAHs in Urban Soil

The descriptive statistics of U.S. EPA sixteen priority PAHs in urban soil in the semi-arid city of Xi’an in Northwest China are given in Table 3. As shown in the table, all sixteen priority PAHs studied were detected in the urban soil, indicating that PAHs were ubiquitous pollutants in the environment. The concentrations of individual PAHs in the urban soil varied from undetected to 1897.6 µg/kg. The total concentration of sixteen PAHs (Σ16PAHs) in the urban soil ranged from 390.6 to 10,652.8 µg/kg with an average of 2052.6 µg/kg. These suggested that PAHs in the urban soil presented a relatively large variation. The total concentrations of seven carcinogenic PAHs (Σ7CPAHs) were in range of 103.9 to 5112.7 µg/kg with a mean of 937.0 µg/kg, averaging 45.7% of Σ16PAHs.

It is very common to compare the concentration levels of ΣPAHs in soil from different cities [13,15,41]. A comparison of ΣPAHs concentrations in soil from different cities worldwide is given in Table 4, where it may be seen that the mean concentration of Σ16PAHs in urban soil of Xi’an was lower than that in urban soil from some other Chinese cities such as Beijing [9,11,58], Nanjing [2], and Shanghai [10] as well as Dhanbad (India) [59], London (UK) [60], New Orleans (USA) [61], and Lisbon (Portugal) [62]. It was comparable with that in urban soil in Lanzhou (China) [1], Bratislava (Slovakia) [63], and Isfahan (Iran) [40]. However, it was higher than that in urban soil from some Chinese cities such as Beijing [41,64,65], Dalian [12], Hong Kong [66], and Shanghai [14,15,67] as well as from Sevilla (Spain) [68], Kragujevac (Serbia) [69], Kumasi (Ghana) [3], Kathmandu (Nepal) [7], Lisbon and Viseu (Portugal) [8], Ulsan (South Korea) [70], and San Mateo Ixtatan (Guatemala) [71]. The concentration comparison showed that PAHs in urban soil of Xi’an corresponded to a moderate level.

### 3.2. Pollution of PAHs in Urban Soil

The concentrations of PAHs in soil have not yet been limited in China. Meanwhile, few recommendations or guidelines for soil PAHs are available in the world. In this study, the Dutch Target Values of Soil Quality [55] was used to compare with the present concentration of some individual PAHs in the urban soil for obtaining pollution levels. The concentrations of NaP, Phe, Ant, BaA, Chy, BkF, BaP, BghiP, and InP in 21, 49, 23, 46, 52, 49, 41, 55, and 37 soil samples were higher than the Dutch Target Values of Soil Quality, respectively, which were 15, 50, 50, 20, 20, 25, 25, 20, and 25 µg/kg [55], respectively. The concentrations of Fla in all soil samples were greater than the Dutch Target Value of Soil Quality, which is 15 µg/kg [55]. In addition, a soil contamination classification standard on the basis of the Σ16PAHs was proposed by Maliszewska-Kordybach [72]. PAHs presented non-contamination with the Σ16PAHs of <200 µg/kg, slight contamination 200–600 µg/kg, medium contamination 600–1000 µg/kg, and heavy contamination >1000 µg/kg. According to this classification standard, 44 soil samples were heavily polluted by PAHs, 13 soil samples were moderately contaminated, and five soil samples were slightly polluted. Overall, PAHs in the urban soil represented heavy pollution.

### 3.3. Ecological Risk of PAHs in Urban Soil

The assessment results of ecological risk of PAHs in the urban soil based on risk quotient are given in Table 5, where the mean values of calculated RQ_(NCs)_ and RQ_(MPCs)_ for Pyr and BDA are above 1, indicating that they present a high ecological risk to aquatic/soil organisms. The average values of calculated RQ_(NCs)_ for other PAHs were greater than 1, while the mean values of calculated RQ_(MPCs)_ for them were lower than 1, implying that other PAHs had moderately ecological risk to aquatic/soil organisms. The mean value of calculated RQ_ΣPAHs(NCs)_ was below 800, while the average of calculated RQ_ΣPAHs(MPCs)_ was higher than 1, suggesting that the total ecological risk of PAHs in urban soil to aquatic/soil organisms was moderate levels. From the ecological risk of individual PAHs and ΣPAHs in the urban soil in urban functional areas of Xi’an (Table 6), Pyr had high ecological risk in urban functional areas except in mixed commercial and traffic areas and residential areas (moderate); BaA presented high ecological risk in industrial areas, while moderate ecological risk in other functional areas; the ecological risk levels of Chy were moderate in the first and third ring roads as well as industrial, traffic and educational areas, while low in other functional areas; the levels of InP and BghiP were moderate ecological risk in urban functional areas except in mixed commercial and traffic areas and residential areas (low); DBA showed high ecological risk in urban functional areas except in the first ring road and industrial areas (moderate); and the ecological risk levels of other individual PAHs were moderate in urban functional areas. The total ecological risk levels of ΣPAHs were high in the second to third ring roads as well as industrial and traffic areas, while moderate in other urban functional areas.

### 3.4. Toxicity Potential of PAHs in Urban Soil

Characterized by the high toxicity, potential carcinogenic, teratogenic, and mutagenic effects, as well as endocrine disruptive activities, PAHs have received more concern. In this study, toxic equivalence factors (TEFs) [46,47] were used to calculate toxic equivalence quantities (TEQs) of PAHs in urban soil of Xi’an for quantifying the toxic potential of PAHs and further evaluating the health risk of human exposure to PAHs. As shown in Table 7, the TEQs of sixteen PAHs in the urban soil ranged from 21.16 to 1625.78 μg/kg with an average of 423.86 μg/kg. The TEQs of seven carcinogenic PAHs in the urban soil varied between 20.63 and 1610.04 μg/kg with an average of 421.05 μg/kg. The TEQs of seven carcinogenic PAHs were very close to that of sixteen PAHs, indicating that the seven carcinogenic PAHs were the main contributor to the TEQs of sixteen PAHs. The contribution of seven carcinogenic PAHs to the TEQs of sixteen PAHs decreased in the order of DBA (66.3%) >> BaP (23.0%) >> BaA (2.9%) > BbF (2.6%) > BkF (2.4%) > InP (1.8%) > Chy (0.4%). The present results were similar to that of urban surface dust: BaP (45%) > DBA (33%) ≫ BbF (5.7%) > InP (5.0%) > BkF (4.9%) > BaA (4.1%) ≫ Chy (0.8%) [32]. The TEQs of sixteen PAHs and seven carcinogenic PAHs in 13 soil samples exceeded the safe level of 600 µg/kg based on the risk-based soil criterion for protection of human health from Canadian Council of Ministers of the Environment [73]. In addition, the TEQs of sixteen PAHs and seven carcinogenic PAHs in urban soil of Xi’an was larger than that in urban soil from Beijing (range 0.7–3240 µg/kg, mean 180.7 µg/kg [9]; mean 27.75 µg/kg [13]), Shanghai (range: 7.02–869 µg/kg, mean 236 µg/kg [14]; range 1.1–620 µg/kg [15]), Lanzhou (range 5.93–1290 µg/kg, mean 136 µg/kg [1]), Isfahan (Iran; range 1.00–900.53 µg/kg, mean 67.39 µg/kg [40]), Lisbon and Viseu (Portugal; mean 229 and 24 µg/kg [8]), and Bratislava (Slovakia; range 7.4–2602 µg/kg, mean 376 µg/kg [63]). It was only lower than that in urban soil from Nanjing of China (mean 445 µg/kg [2]) and Dhanbad (India; mean 720 µg/kg [59]). These indicated that PAHs in urban soil of Xi’an presented relatively high toxicity potency.

### 3.5. Health Risk of PAHs in Urban Soil

As shown in Table 8, the cancer risk levels of human exposure to PAHs in urban soil through ingestion and dermal adsorption ranged from 10^−7^ to 10^−5^, which were 10^4^ to 10^5^ times higher than that through inhalation. Thus, inhalation of soil dust relative to ingestion and dermal adsorption of soil dust was negligible. Similar results were observed in human exposure to heavy metals from dust in an industrial area of Baoji, to phthalic acid esters in street dust of Xi’an, and to PAHs from urban surface dust of Xi’an as well as from urban soil of Isfahan (Iran) and Shanghai (China) [15,32,40,74,75].

The cancer risk levels through ingestion for children and adults were on the same order of magnitude (10^−7^ to 10^−5^) as through dermal adsorption, indicating that ingestion and dermal adsorption mainly contributed to the cancer risk to children and adults. However, the risk values of ingestion for children were higher than the corresponding risk of ingestion for adults. Generally, children are the most sensitive subpopulation because of their more hand-to-mouth activities relative to adults. Thus, contaminated soil/dust in the urban environment can be readily ingested [15,39,40,45,76]. In addition, the PAH intake by a child is believed to be greater than that by an adult without raining health effects because children have lower body weights relative to adults. Therefore, the health risks of children exposure to PAHs from urban soil/dust are considerably greater than those of adults [15,45]. The health risk levels of adults through inhalation and dermal contact were greater than those for children. Similar results were found in human exposure to PAHs from urban soil of Beijing and Shanghai [15,41], from urban surface dust of Guangzhou [45], and from street dust of Lanzhou [39], which could be related to the higher values of inhalation rate, dermal exposure area, and exposure duration for adults [15,39,40,45].

The potential cancer risk is under the acceptance range with an ILCR value of 10^−6^ to 10^−4^, low or negligible below 10^−6^, and a high cancer risk above 10^−4^ [15,38,39,40,45]. In this study, the 95% confidence intervals of ILCRs for total cancer risk were 2.86 × 10^−5^ for children and 2.53 × 10^−5^ for adults, respectively, which are in the range of 10^−6^ to 10^−4^. These values show that the total cancer risk from human exposure to PAHs from urban soil of Xi’an is acceptable. Meanwhile, human exposure to PAHs poses health risk via multimedia and multi-pathway. Wang et al. [32] reported that the ILCR values of human exposure to PAHs from urban surface dust of Xi’an are 8.2 × 10^−5^ for children and 7.3 × 10^−5^ for adults, respectively, which are on the same order of magnitude as those in the urban soil in the present study. These values indicate that the cancer risk of human exposure to PAHs from urban soil is comparable to that from urban surface dust. 

**Figure 2:**
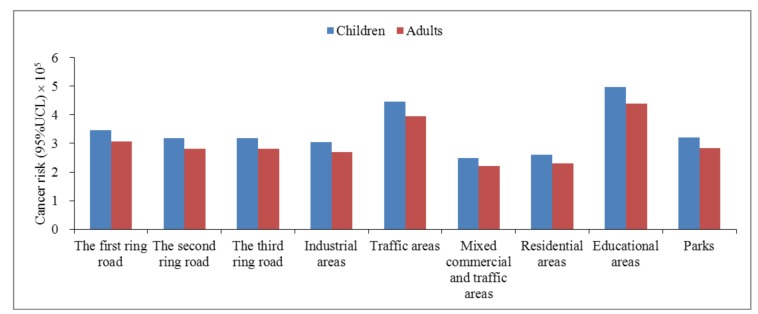
Cancer risk of human exposure to PAHs from urban soil in urban functional areas of Xi’an.

As shown in Figure 2, the total cancer risk of children and adults exposure to PAHs from urban soil is relatively high in educational and traffic areas, followed by the first to third ring roads, industrial areas and parks, and relatively low in mixed commercial and traffic areas as well as residential areas. In addition, they decrease from the first to third ring roads. Therefore, more attention should be paid to educational and traffic areas.

## 4. Conclusions

PAHs are ubiquitous environmental pollutants, posing potential threats to ecological environment and human health. Sixty-two urban soil samples were collected in the typical semi-arid city of Xi’an in Northwest China. They were analyzed for pollution level as well as ecological and health risk of sixteen PAHs from the U.S. EPA priority list. The results showed that all sixteen priority PAHs were detected in urban soil. The total concentrations of sixteen PAHs (Σ16PAHs) ranged from 390.6 to 10652.8 µg/kg with an average of 2052.6 µg/kg, belonging to the moderate level. The concentrations of some individual PAHs exceeded the Dutch Target Values of Soil Quality to different degree. Overall, the Σ16PAHs presented heavy pollution. Pyr and DBA had high ecological risk to aquatic/soil organisms, while other PAHs presented low ecological risk. The total ecological risk of PAHs to aquatic/soil organisms was moderate. TEQs of sixteen PAHs in urban soil of Xi’an ranged from 21.16 to 1625.78 µg/kg with a mean of 423.86 µg/kg, and had relatively high toxicity potency resulting mainly from seven carcinogenic PAHs. Ingestion and dermal adsorption of soil dust were the major pathways of human exposure to PAHs from urban soil. The risk level of children exposure to PAHs from urban soil through ingestion of soil dust was higher than that of adults, while the level of children exposure via inhalation and dermal adsorption was lower than that of adults. The ILCRs for children and adults were 2.86 × 10^−5^ and 2.53 × 10^−5^, respectively, which being in the range of 10^−6^ to 10^−4^, suggesting the cancer risk of human exposure to PAHs from urban soil acceptable.

## Figures and Tables

**Table 1:** Ecological risk classification of individual and total PAHs.

Individual PAHs			ΣPAHs		
	RQ_(NCs)_	RQ_(MPCs)_		RQ_ΣPAHs(NCs)_	RQ_ΣPAHs(MPCs)_
Risk-free	0		Risk-free	=0	
			Low-risk	≥1; <800	=0
Moderate-risk	≥1	<1	Moderate-risk1	≥800	=0
			Moderate-risk2	<800	≥1
High-risk		≥1	High-risk	≥800	≥1

**Table 2:** Values of parameters used in health risk assessment models.

Parameters	Unit	Children	Adults	References
Ingestion rate (IngR)	mg/day	200	100	[48]
Exposure frequency (EF)	day/year	180		[49]
Exposure duration (ED)	year	6	24	[48]
Body weight (BW)	kg	15	58.6	[48,50,51]
Average time (AT)	day	Carcinogen 70 × 365 = 2550		[49]
Inhalation rate (InhR)	m^3^/day	7.6	12.8	[50,51,52]
Particle emission factor (PEF)	m^3^/kg	1.36 × 10^9^	1.36 × 10^9^	[48]
Skin surface area (SA)	cm^2^	1150	2145	[50,53]
Skin adherence factor (AF)	mg/cm^2^	0.2	0.07	[54]
Adsorption factor (ABS)	unitless	0.13	0.13	[48,54]

**Table 3:** Concentration of PAHs in urban soil of Xi’an (µg/kg).

PAHs	Min	Max	Mean	SD	CV	AC
Nap	ND	140.3	16.8	20.2	0.83	15
Acy	32.9	538.7	63.3	66.9	0.95	
Ace	24.3	523.5	62.0	68.8	0.90	
Flu	10.3	477.3	63.0	95.4	0.66	
Phe	11.0	1633.4	145.2	231.8	0.63	50
Ant	18.1	1090.9	78.7	143.3	0.55	50
Fla	56.8	1303.0	351.8	280.0	1.26	15
Pyr	21.7	1697.4	225.4	381.0	0.59	
BaA	5.9	1897.6	122.4	274.0	0.45	20
Chy	ND	1620.3	147.6	274.0	0.54	20
BbF	ND	1026.2	110.2	170.0	0.65	
BkF	ND	878.4	100.4	151.7	0.66	25
BaP	ND	938.3	97.3	186.3	0.52	25
InP	ND	871.0	77.9	136.2	0.57	25
DBA	ND	1477.8	281.2	273.5	1.03	
BghiP	ND	1116.9	109.5	175.6	0.62	20
Σ16PAHs	390.6	10652.8	2052.6	2207.6	0.93	
Σ7CPAHs	103.9	5112.7	937.0	1065.2	0.88	
ΣCOMB	149.2	8183.4	1342.5	1783.6	0.75	
LMWPAHs	121.0	4288.5	429.0	587.1	0.73	
HMWPAHs	214.6	8618.6	1623.7	1812.0	0.90	

ND: not detected; SD: standard deviation; CV: coefficient of variation; AC: acceptable concentrations [55]; Σ16PAHs: the sum of sixteen individual PAHs; Σ7CPAHs: the total of seven carcinogenic PAHs including BaA, Chy, BbF, BkF, BaP, DBA, and InP; ΣCOMB: the sum of major combustion-specific compounds containing Fla, Pyr, BaA, Chy, BbF, BkF, BaP, BghiP, and InP [44,56,57]; LMWPAHs: the total of low molecular weight PAHs, i.e, NaP, Acy, Ace, Flu, Phe, and Ant; HMWPAHs: the sum of high molecular weight PAHs, i.e, Fla, Pyr, BaA, Chy, BbF, BkF, BaP, DBA, BghiP, and InP.

**Table 4:** Concentration comparison of ΣPAHs in soil from worldwide different cities (µg/kg).

Cities	Number of PAHs	Soil Types	Min	Max	Mean	References
Xi’an, China	16	urban soil	390.6	10,652.8	2052.6	In this study
Beijing, China	16	urban soil	16	3884	1347	[64]
Beijing, China	16	urban soil	366	27,825	3917	[58]
Beijing, China	16	urban soil	467	5470	1637	[65]
Beijing, China	15	urban soil	112	27,800	6440	[11]
Beijing, China	16	urban soil	8.5	13,126	1802.6	[9]
Beijing, China	16	urban soil	93	13,141	1228	[41]
Dalian, China	14	urban, suburban and rural soil	219	18,727	1104	[12]
Hong Kong, China	16	urban soil	42.9	410	169	[66]
Lanzhou, China	16	urban soil	82.2	10,900	2360	[1]
Nanjing, China	16	urban soil	56.8	18,000	3330	[2]
Shanghai, China	16	urban soil	347	17,900	3290	[10]
Shanghai, China	16	urban soil	62.4	31,900	1700	[67]
Shanghai, China	16	urban soil	83.3	7220	1970	[14]
Shanghai, China	16	urban and suburban soil	18.8	6320	807	[15]
Bratislava, Slovakia	16	urban soil	45	12,151	2064.8	[63]
Dhanbad, India	13	urban traffic soil	1019	10,856	3488	[59]
Isfahan, Iran	16	urban soil	57.70	11,730.08	2000.56	[40]
Kragujevac, Serbia	15	urban soil	38	3136	240	[69]
Kumasi, Ghana	22	urban soil	14.78	2084	442.5	[3]
Kathmandu, Nepal	20	urban soil	184	10,279	1556	[7]
Lisbon, Portugal	16	urban soil	6	73,395	2717	[62]
Lisbon, Portugal	16	urban soil	6.3	22,670	1544	[8]
London, UK	16	urban soil	4000	67,000	18,000	[60]
New Orleans, USA	16	urban soil			2927	[61]
Sevilla, Spain	15	urban soil	89.5	4004.2	810.2	[68]
Ulsan, South Korea	16	urban, industrial and rural soil	65	12,000	960	[70]
Viseu, Portugal	16	urban soil	6.0	790	169	[8]
San Mateo Ixtatan	17	urban and peri-urban soil	460	3251	1401	[71]

**Table 5:** Descriptive statistics of RQ_(NCs)_ and RQ_(MPCs)_ of PAHs in urban soil (µg/kg).

PAHs	NCs	MPCs	RQ_(NCs)_					RQ_(MPCs)_				
			Min	Max	Mean	SD	CV	Min	Max	Mean	SD	CV
Nap	1.4	1400	0.00	100.23	11.97	14.43	0.83	0.00	1.00	0.12	0.14	0.83
Acy	1.2	1200	27.43	448.88	52.74	55.74	0.95	0.27	4.49	0.53	0.56	0.95
Ace	1.2	1200	20.27	436.25	51.69	57.30	0.90	0.20	4.36	0.52	0.57	0.90
Flu	1.2	1200	8.57	397.72	52.47	79.48	0.66	0.09	3.98	0.52	0.79	0.66
Phe	5.1	5100	2.16	320.27	28.47	45.44	0.63	0.02	3.20	0.28	0.45	0.63
Ant	1.2	1200	15.07	909.10	65.61	119.39	0.55	0.15	9.09	0.66	1.19	0.55
Fla	26	26,000	2.18	50.11	13.53	10.77	1.26	0.02	0.50	0.14	0.11	1.26
Pyr	1.2	1200	18.08	1414.51	187.85	317.49	0.59	0.18	14.15	1.88	3.17	0.59
BaA	2.5	2500	2.36	759.05	48.96	109.58	0.45	0.02	7.59	0.49	1.10	0.45
Chy	107	107,000	0.00	15.14	1.38	2.56	0.54	0.00	0.15	0.01	0.03	0.54
BbF	2.5	2500	0.00	410.49	44.08	67.99	0.65	0.00	4.10	0.44	0.68	0.65
BkF	24	24,000	0.00	36.60	4.18	6.32	0.66	0.00	0.37	0.04	0.06	0.66
BaP	2.6	2600	0.00	360.89	37.44	71.66	0.52	0.00	3.61	0.37	0.72	0.52
InP	59	59,000	0.00	14.76	1.32	2.31	0.57	0.00	0.15	0.01	0.02	0.57
DBA	2.6	2600	0.00	568.37	108.14	105.19	1.03	0.00	5.68	1.08	1.05	1.03
BghiP	75	75,000	0.00	14.89	1.46	2.34	0.62	0.00	0.15	0.01	0.02	0.62
ΣPAHs			183.51	4769.68	710.40	804.44	0.88	0.00	45.97	4.21	8.20	0.51

SD: standard deviation; CV: coefficient of variation.

**Table 6:** Ecological risk of individual and total PAHs in the urban soil in urban functional areas in Xi’an.

Functional Areas	Nap	Acy	Ace	Flu	Phe	Ant	Fla	Pyr	BaA	Chy	BbF	BkF	BaP	InP	DBA	BghiP	ΣPAHs
The first ring road	M	M	M	M	M	M	M	H	M	M	M	M	M	M	M	M	M2
The second ring road	M	M	M	M	M	M	M	H	M	L	M	M	M	M	H	M	M2
The third ring road	M	M	M	M	M	M	M	H	M	M	M	M	M	M	H	M	H
Industrial areas	M	M	M	M	M	M	M	H	H	M	M	M	M	M	M	M	H
Traffic areas	M	M	M	M	M	M	M	H	M	M	M	M	M	M	H	M	H
Mixed commercial and traffic areas	M	M	M	M	M	M	M	M	M	L	M	M	M	L	H	L	M2
Residential areas	M	M	M	M	M	M	M	M	M	L	M	M	M	L	H	L	M2
Educational areas	M	M	M	M	M	M	M	H	M	M	M	M	M	M	H	M	M2
Parks	M	M	M	M	M	M	M	H	M	L	M	M	M	M	H	M	M2

L: risk-free; M: moderate risk; H: high risk.

**Table 7:** Toxic equivalence quantities (TEQs) of PAHs in urban soil in Xi’an (µg/kg).

PAHs	TEFs	Min	Max	Mean	SD	CV
Nap	0.001	ND	0.14	0.02	0.02	0.83
Acy	0.001	0.03	0.54	0.06	0.07	0.95
Ace	0.001	0.02	0.52	0.06	0.07	0.90
Flu	0.001	0.01	0.48	0.06	0.10	0.66
Phe	0.001	0.01	1.63	0.15	0.23	0.63
Ant	0.01	0.18	10.91	0.79	1.43	0.55
Fla	0.001	0.06	1.30	0.35	0.28	1.26
Pyr	0.001	0.02	1.70	0.23	0.38	0.59
BaA	0.1	0.59	189.76	12.24	27.40	0.45
Chy	0.01	ND	16.20	1.48	2.74	0.54
BbF	0.1	ND	102.62	11.02	17.00	0.65
BkF	0.1	ND	87.84	10.04	15.17	0.66
BaP	1.0	ND	938.31	97.33	186.30	0.52
InP	0.1	ND	87.10	7.79	13.61	0.57
DBA	1.0	ND	1477.77	281.16	273.50	1.03
BghiP	0.01	ND	11.17	1.09	1.76	0.62
16PAHs		21.16	1625.78	423.86	363.65	1.17
7CPAHs		20.63	1610.04	421.05	361.80	1.16

ND: not detected; TEFs: toxic equivalency factors [46,47]; SD: standard deviation; CV: coefficient of variation.

**Table 8:** Health risk of human exposure to PAHs in urban soil.

Items	CS	Children				Adults			
	mg/kg	Ingestion	Inhalation	Dermal	Cancer Risk	Ingestion	Inhalation	Dermal	Cancer Risk
Min	2.12 × 10^−2^	5.22 × 10^−7^	7.69 × 10^−12^	6.51 × 10^−7^	1.17 × 10^−6^	3.74 × 10^−7^	2.90 × 10^−11^	6.64 × 10^−7^	1.04 × 10^−6^
Max	1.63 × 10^0^	4.01 × 10^−5^	5.91 × 10^−10^	5.00 × 10^−5^	9.01 × 10^−5^	2.87 × 10^−5^	2.23 × 10^−9^	5.10 × 10^−5^	7.97 × 10^−5^
Mean	4.24 × 10^−1^	1.05 × 10^−5^	1.54 × 10^−10^	1.30 × 10^−5^	2.35 × 10^−5^	7.49 × 10^−6^	5.81 × 10^−10^	1.33 × 10^−5^	2.08 × 10^−5^
Median	3.12 × 10^−1^	7.70 × 10^−6^	1.13 × 10^−10^	9.59 × 10^−6^	1.73 × 10^−5^	5.51 × 10^−6^	4.28 × 10^−10^	9.79 × 10^−6^	1.53 × 10^−5^
95%LCL	3.32 × 10^−1^	8.18 × 10^−6^	1.21 × 10^−10^	1.02 × 10^−5^	1.84 × 10^−5^	5.86 × 10^−6^	4.54 × 10^−10^	1.04 × 10^−5^	1.63 × 10^−5^
95%UCL	5.16 × 10^−1^	1.27 × 10^−5^	1.88 × 10^−10^	1.59 × 10^−5^	2.86 × 10^−5^	9.12 × 10^−6^	7.07 × 10^−10^	1.62 × 10^−5^	2.53 × 10^−5^

CS: the sum of toxic equivalency quantities (TEQs) of sixteen PAHs relative to BaP using the toxic equivalency factors (TEFs) listed in Table 7 [46,47].
